# Rapid chromosomal evolution in enigmatic mammal with XX in both sexes, the Alay mole vole *Ellobiusalaicus* Vorontsov et al., 1969 (Mammalia, Rodentia)

**DOI:** 10.3897/CompCytogen.v13i2.34224

**Published:** 2019-06-20

**Authors:** Irina Bakloushinskaya, Elena A. Lyapunova, Abdusattor S. Saidov, Svetlana A. Romanenko, Patricia C.M. O’Brien, Natalia A. Serdyukova, Malcolm A. Ferguson-Smith, Sergey Matveevsky, Alexey S. Bogdanov

**Affiliations:** 1 Koltzov Institute of Developmental Biology, Russian Academy of Sciences, Moscow, Russia Koltzov Institute of Developmental Biology, Russian Academy of Sciences Moscow Russia; 2 Pavlovsky Institute of Zoology and Parasitology, Academy of Sciences of Republic of Tajikistan, Dushanbe, Tajikistan Pavlovsky Institute of Zoology and Parasitology, Academy of Sciences of Republic of Tajikistan Dushanbe Tajikistan; 3 Institute of Molecular and Cellular Biology, Siberian Branch RAS, Novosibirsk, Russia Institute of Molecular and Cellular Biology, Siberian Branch of Russian Academy of Sciences Novosibirsk Russia; 4 Novosibirsk State University, Novosibirsk, Russia Novosibirsk State University Novosibirsk Russia; 5 Cambridge Resource Centre for Comparative Genomics, Department of Veterinary Medicine, University of Cambridge, Cambridge, UK University of Cambridge Cambridge United Kingdom; 6 Vavilov Institute of General Genetics, Russian Academy of Sciences, Moscow, Russia Vavilov Institute of General Genetics, Russian Academy of Sciences Moscow Russia

**Keywords:** speciation, hybridization, chromosome painting, cytochrome *b* gene, nuclear *XIST* and *Rspo1* genes, Robertsonian translocations, synaptonemal complex, *
Ellobius
*

## Abstract

Evolutionary history and taxonomic position for cryptic species may be clarified by using molecular and cytogenetic methods. The subterranean rodent, the Alay mole vole *Ellobiusalaicus* Vorontsov et al., 1969 is one of three sibling species constituting the subgenus Ellobius Fischer, 1814, all of which lost the Y chromosome and obtained isomorphic XX sex chromosomes in both males and females. *E.alaicus* is evaluated by IUCN as a data deficient species because their distribution, biology, and genetics are almost unknown. We revealed specific karyotypic variability (2n = 52–48) in *E.alaicus* due to different Robertsonian translocations (Rbs). Two variants of hybrids (2n = 53, different Rbs) with *E.tancrei* Blasius, 1884 were found at the Northern slopes of the Alay Ridge and in the Naryn district, Kyrgyzstan. We described the sudden change in chromosome numbers from 2n = 50 to 48 and specific karyotype structure for mole voles, which inhabit the entrance to the Alay Valley (Tajikistan), and revealed their affiliation as *E.alaicus* by cytochrome *b* and fragments of nuclear *XIST* and *Rspo1* genes sequencing. To date, it is possible to expand the range of *E.alaicus* from the Alay Valley (South Kyrgyzstan) up to the Ferghana Ridge and the Naryn Basin, Tien Shan at the north-east and to the Pamir-Alay Mountains (Tajikistan) at the west. The closeness of *E.tancrei* and *E.alaicus* is supported, whereas specific chromosome and molecular changes, as well as geographic distribution, verified the species status for *E.alaicus*. The case of *Ellobius* species accented an unevenness in rates of chromosome and nucleotide changes along with morphological similarity, which is emblematic for cryptic species.

## Introduction

An origin of species due to chromosome changes is still debatable (King 1993, Castiglia 2014, Dobigny et al. 2017). The problem of chromosomal speciation is closely connected with the phenomenon of sibling species. Mole voles of the genus *Ellobius* Fischer, 1814, and some other rodents, such as *Mus* Linnaeus, 1758, *Nannomys* Peters, 1876 (Gropp et al. 1972, Capanna et al. 1976, Capanna and Castiglia 2004, Veyrunes et al. 2010, Garagna et al. 2014), and subterranean *Spalax* Guldenstaedt, 1770, *Fukomys* Kock et al., 2006, *Ctenomys* Blainville, 1826 etc. (Wahrman et al. 1969, Nevo et al. 2000, Van Daele et al. 2007, Deuve et al. 2008, Kryštufek et al. 2012, Buschiazzo et al. 2018), demonstrate a broad chromosome variability at the species and intraspecies levels without morphological differences (Lyapunova et al. 1980). The lack of clear morphological characters, by which specimens can be easily distinguished in museum collections, as well as in nature, makes such species problematic for study and protection. New molecular methods, especially DNA sequencing and cross-species chromosome painting, can be a precise approach for studying the most intriguing groups (Graphodatsky et al. 2011).

The genus *Ellobius* divides into two subgenera: *Bramus* Pomel, 1892 and *Ellobius* Fischer, 1814 (Musser, Carleton 2005). The subgenus Bramus includes two species: *E.fuscocapillus* Blyth, 1843 (2n = 36, XX♀–XY♂), and *E.lutescens* Thomas, 1897 (2n = 17, X0♀-X0♂) (Matthey 1953, Vorontsov et al. 1969, Lyapunova, Vorontsov 1978). Species of the subgenus Ellobius (*E.talpinus* Pallas, 1770, *E.tancrei* Blasius, 1884, and *E.alaicus*Vorontsov et al. 1969) are cryptic ones, indistinguishable by morphological features (Yakimenko and Vorontsov 1982), the main diagnostic features are distant karyotypes. *E.talpinus*, *E.tancrei*, and *E.alaicus* are unique in mammals. Along with autosomal changes, the species lost the Y chromosome, the *Sry* gene, and obtained isomorphic XX chromosomes in both males and females (Lyapunova and Vorontsov 1978, Vorontsov et al. 1980, Kolomiets et al. 1991, Just et al. 1995, Romanenko et al. 2007, Bakloushinskaya et al. 2012, Bakloushinskaya and Matveevsky 2018). The study of *E.lutescens* and *E.talpinus* whole genomes was not able to reveal any sex determining factors (Mulugeta et al. 2016). The first signs of sex chromosomes heteromorphism in *E.talpinus* and *E.tancrei* were observed in the meiotic behaviour of XX chromosomes in males ([Bibr B37][Bibr B36], [Bibr B49][Bibr B51]).

The northern mole vole, *E.talpinus*, with 2n = NF = 54 (Ivanov 1967, Romanenko et al. 2007), has no described chromosomal variability, but significant mtDNA variability was revealed recently along its wide range (Bogdanov et al. 2015). The eastern mole vole, *E.tancrei* has stable 2n = 54, NF = 56 in most of its range, and demonstrates enormous karyotype variability (2n = 54-30) in the Pamir-Alay region (Vorontsov and Radzhabli 1967, [Bibr B44][Bibr B42], Bakloushinskaya et al. 2013). The third species was described first as a chromosomal form of *E.talpinus* sensu lato (a chromosomal form of *E.tancrei* from the modern point of view) with one pair of large metacentric chromosomes and small submetacentrics, specific 2n = 52, NF = 56 (Vorontsov and Radzhabli 1967), and later it was designated as the Alay mole vole *E.alaicus* (Vorontsov et al. 1969, Lyapunova and Vorontsov 1978). The Alay Valley, the *terra typica* of the Alay mole vole, extending appr. 180 km from Tajikistan in the west to China in the east between two mountain systems: the Tien Shan and the Pamir. Range of the species was limited to the Alay Valley and the Northern slopes of the Alay Ridge, Tien-Shan (Kyrgyzstan). *E.alaicus* was listed by IUCN as data deficient species; cytogenetic data are scarce, no molecular study has been made ever (Gerrie and Kennerley 2016).

We studied the G-band structure of the *E.alaicus* karyotype previously and described a morphological homology for one pair of large metacentrics of the species to the Robertsonian metacentrics of *E.tancrei* from the Pamir-Alay (Bakloushinskaya 2003). We also discovered different forms of *E.alaicus* and their hybrids with *E.tancrei* with 2n = 50-53 from other parts of the Inner Tien-Shan (Lyapunova et al. 1985, Bakloushinskaya, Lyapunova 2003). But the study was incomplete, and application of modern cytogenetical and molecular techniques is required to confirm the karyotype structure, validity of *E.alaicus* as a species and its distribution.

The main objectives of this study were to reveal the chromosomal variability in *E.alaicus* and prove species affiliations for mole voles from adjacent to the Alay Valley territories of the Inner Tien-Shan and the Pamir-Alay Mountains. To bring a phylogenetic framework to the delimiting species, we examined the phylogeny of the subgenus Ellobius using the mitochondrial DNA marker, complete cytochrome *b* gene, *cytb*, and two nuclear DNA markers, fragments of the *XIST* (X-inactive specific transcript) and *Rspo1* (R-spondin 1) genes.

## Material and methods

**Figure 1.**
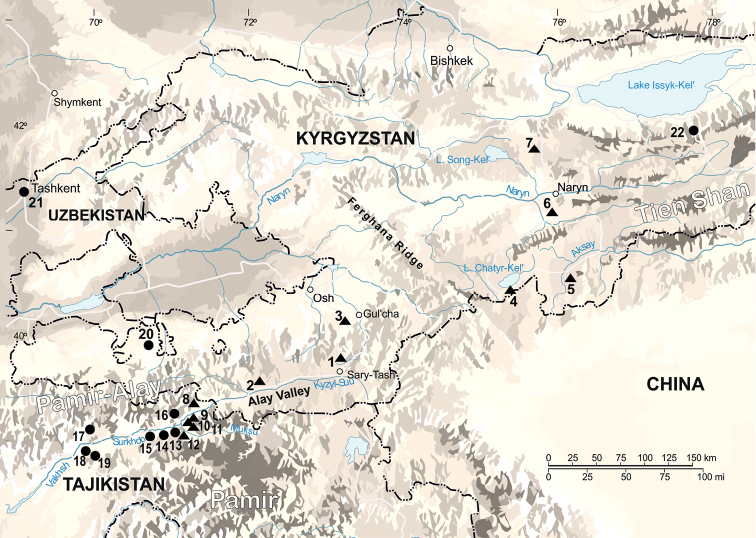
The geographic location of studied populations of the mole voles *E.alaicus* (dark triangles) and *E.tancrei* (dark spots). Localities are numbered as in Table 1. Localities 23–27 are outside the map.

We analyzed karyotypes or *cytb* structure, or both, of 116 specimens of *E.alaicus* and *E.tancrei* mole voles from 27 localities across the Alay Valley and adjacent territories, as well as 7 *E.talpinus* specimens from 6 localities of Russia (Fig. 1, Table 1). Fragments of the *XIST* and *Rspo1* genes were studied for nine specimens of three species.

**Table 1.** List of studied specimens, species, origin/locality, sex, 2n, *cytb* accession numbers.

No	Species	2n	Voucher #	Sex	Loc. #	Locality	Coordinates	Year	GenBank #
1	* E. alaicus *	–	S132131*	♂	1	Kyrgyzstan. The Alay Valley, 10 km to the North from the Sary-Tash, the Taldyk pass, 3500 m above sea level	39°46'N 73°10'E	1983	MG264319
2	* E. alaicus *	–	S132133*	♀	1	Kyrgyzstan. The Alay Valley, 10 km to the North from the Sary-Tash, the Taldyk pass, 3500 m above sea level	39°46'N 73°10'E	1983	MG264320
3	* E. alaicus *	–	S132135*	♀	1	Kyrgyzstan. The Alay Valley, 10 km to the North from the Sary-Tash, the Taldyk pass, 3500 m above sea level	39°46'N 73°10'E	1983	MG264321
4	* E. alaicus *	–	S132130*	♂	2	Kyrgyzstan. The Alay Valley, close to Daraut-Korgon settlement, 2160 m above sea level	39°33'N 72°15'E	1983	MG264318
5	*E.alaicus* × *E.tancrei* hybrid	53	20757	♂	3	Kyrgyzstan. Pamir Highway, Osh – Gul’cha. 20 km to Gul’cha, the beginning of the ascent to the pass, 1500 m above sea level	40°15'N 73°20'E	1983	–
6	* E. alaicus *	52	20758	♂	3	Kyrgyzstan. Pamir Highway, Osh – Gul’cha. 20 km to Gul’cha, the beginning of the ascent to the pass, 1500 m above sea level	40°15'N 73°20'E	1983	–
7	*E.alaicus* × *E.tancrei* hybrid	53	20759	♂	3	Kyrgyzstan. Pamir Highway, Osh – Gul’cha. 20 km to Gul’cha, the beginning of the ascent to the pass, 1500 m above sea level	40°15'N 73°20'E	1983	–
8	* E. alaicus *	52	20760	♂	3	Kyrgyzstan. Pamir Highway, Osh – Gul’cha. 20 km to Gul’cha, the beginning of the ascent to the pass, 1500 m above sea level	40°15'N 73°20'E	1983	–
9	* E. alaicus *	52	20764	♂	3	Kyrgyzstan. Pamir Highway, Osh – Gul’cha. 20 km to Gul’cha, the beginning of the ascent to the pass, 1500 m above sea level	40°15'N 73°20'E	1983	–
10	* E. alaicus *	52	20765	♀	3	Kyrgyzstan. Pamir Highway, Osh – Gul’cha. 20 km to Gul’cha, the beginning of the ascent to the pass, 1500 m above sea level	40°15'N 73°20'E	1983	–
11	* E. alaicus *	52	20766	♂	3	Kyrgyzstan. Pamir Highway, Osh – Gul’cha. 20 km to Gul’cha, the beginning of the ascent to the pass, 1500 m above sea level	40°15'N 73°20'E	1983	–
12	*E.alaicus* × *E.tancrei* hybrid	53	20778	♂	3	Kyrgyzstan. Pamir Highway, Osh – Gul’cha. 20 km to Gul’cha, the beginning of the ascent to the pass, 1500 m above sea level	40°15'N 73°20'E	1983	–
13	* E. alaicus *	52	20779	♀	3	Kyrgyzstan. Pamir Highway, Osh – Gul’cha. 20 km to Gul’cha, the beginning of the ascent to the pass, 1500 m above sea level	40°15'N 73°20'E	1983	–
14	* E. alaicus *	52	20780	♀	3	Kyrgyzstan. Pamir Highway, Osh – Gul’cha. 20 km to Gul’cha, the beginning of the ascent to the pass, 1500 m above sea level	40°15'N 73°20'E	1983	–
15	* E. alaicus *	52	20788	♂	3	Kyrgyzstan. Pamir Highway, Osh – Gul’cha. 20 km to Gul’cha, the beginning of the ascent to the pass, 1500 m above sea level	40°15'N 73°20'E	1983	–
16	* E. alaicus *	52	20789	♀	3	Kyrgyzstan. Pamir Highway, Osh – Gul’cha. 20 km to Gul’cha, the beginning of the ascent to the pass, 1500 m above sea level	40°15'N 73°20'E	1983	–
17	* E. alaicus *	52	20790	♀	3	Kyrgyzstan. Pamir Highway, Osh – Gul’cha. 20 km to Gul’cha, the beginning of the ascent to the pass, 1500 m above sea level	40°15'N 73°20'E	1983	–
18	* E. alaicus *	52	20791	♂	3	Kyrgyzstan. Pamir Highway, Osh – Gul’cha. 20 km to Gul’cha, the beginning of the ascent to the pass, 1500 m above sea level	40°15'N 73°20'E	1983	–
19	* E. alaicus *	52	20792	♂	3	Kyrgyzstan. Pamir Highway, Osh – Gul’cha. 20 km to Gul’cha, the beginning of the ascent to the pass, 1500 m above sea level	40°15'N 73°20'E	1983	–
20	* E. alaicus *	52	21054	♂	4	Kyrgyzstan. Close to the lake Chatyr-Kel', the 522 km from Bishkek city	40°33'N 75°17'E	1983	–
21	* E. alaicus *	51	21055	♀	4	Kyrgyzstan. Close to the lake Chatyr-Kel', the 522 km from Bishkek city	40°33'N 75°17'E	1983	–
22	* E. alaicus *	52	21056	♂	4	Kyrgyzstan. Close to the lake Chatyr-Kel', the 522 km from Bishkek city	40°33'N 75°17'E	1983	–
23	* E. alaicus *	52	21057	♂	4	Kyrgyzstan. Close to the lake Chatyr-Kel', the 522 km from Bishkek city	40°33'N 75°17'E	1983	–
24	* E. alaicus *	52	21058	♀	4	Kyrgyzstan. Close to the lake Chatyr-Kel', the 522 km from Bishkek city	40°33'N 75°17'E	1983	–
25	* E. alaicus *	51	21084	♂	4	Kyrgyzstan. Close to the lake Chatyr-Kel', the 522 km from Bishkek city	40°33'N 75°17'E	1983	–
26	* E. alaicus *	52	21085	♂	4	Kyrgyzstan. Close to the lake Chatyr-Kel', the 522 km from Bishkek city	40°33'N 75°17'E	1983	–
27	* E. alaicus *	51	21086	♀	4	Kyrgyzstan. Close to the lake Chatyr-Kel', the 522 km from Bishkek city	40°33'N 75°17'E	1983	–
28	* E. alaicus *	52	21066	♀	5	Kyrgyzstan. The Aksay River Valley, 4 km to the south-west from the Aksay settlement	40°14'N 73°20'E	1983	–
29	* E. alaicus *	52	21067	♀	5	Kyrgyzstan. The Aksay River Valley, 4 km to the south-west from the Aksay settlement	40°14'N 73°20'E	1983	–
30	* E. alaicus *	52	21083	♀	5	Kyrgyzstan. The Aksay River Valley, 4 km to the south-west from the Aksay settlement	40°14'N 73°20'E	1983	–
31	* E. alaicus *	52	21049	♂	6	Kyrgyzstan. Highway Bishkek - Chatyr-Kel', 362 km	41°21'N 75°59'E	1983	–
32	* E. alaicus *	52	21050	♀	6	Kyrgyzstan. Highway Bishkek - Chatyr-Kel', 362 km	41°21'N 75°59'E	1983	–
33	* E. alaicus *	52	21051	♀	6	Kyrgyzstan. Highway Bishkek - Chatyr-Kel', 362 km	41°21'N 75°59'E	1983	–
34	* E. alaicus *	52	21052	♂	6	Kyrgyzstan. Highway Bishkek - Chatyr-Kel', 362 km	41°21'N 75°59'E	1983	–
35	* E. alaicus *	51	21053	♀	6	Kyrgyzstan. Highway Bishkek - Chatyr-Kel', 362 km	41°21'N 75°59'E	1983	–
36	* E. alaicus *	52	21069	♀	6	Kyrgyzstan. Highway Bishkek - Chatyr-Kel', 362 km	41°21'N 75°59'E	1983	–
37	* E. alaicus *	51	21070	♀	6	Kyrgyzstan. Highway Bishkek - Chatyr-Kel', 362 km	41°21'N 75°59'E	1983	–
38	* E. alaicus *	52	21071	♂	6	Kyrgyzstan. Highway Bishkek - Chatyr-Kel', 362 km	41°21'N 75°59'E	1983	–
39	* E. alaicus *	50	21087	♂	6	Kyrgyzstan. Highway Bishkek - Chatyr-Kel', 362 km	41°21'N 75°59'E	1983	–
40	* E. alaicus *	51	21088	♂	6	Kyrgyzstan. Highway Bishkek - Chatyr-Kel', 362 km	41°21'N 75°59'E	1983	–
41	* E. alaicus *	50	21089	♂	6	Kyrgyzstan. Highway Bishkek - Chatyr-Kel', 362 km	41°21'N 75°59'E	1983	–
42	* E. alaicus *	52	21090	♀	6	Kyrgyzstan. Highway Bishkek - Chatyr-Kel', 362 km	41°21'N 75°59'E	1983	–
43	* E. alaicus *	50	21091	♂	6	Kyrgyzstan. Highway Bishkek - Chatyr-Kel', 362 km	41°21'N 75°59'E	1983	–
44	*E.alaicus* × *E.tancrei* hybrid	53	21059	♀	7	Kyrgyzstan. Highway Bishkek - Chatyr-Kel', 270 km, 4 km after Sary-Bulak settlement	41°55'N 75°43'E	1983	–
45	* E. tancrei *	54	21060	♂	7	Kyrgyzstan. Highway Bishkek - Chatyr-Kel', 270 km, 4 km after Sary-Bulak settlement	41°55'N 75°43'E	1983	–
46	*E.alaicus* × *E.tancrei* hybrid	53	21061	♂	7	Kyrgyzstan. Highway Bishkek - Chatyr-Kel', 270 km, 4 km after Sary-Bulak settlement	41°55'N 75°43'E	1983	–
47	* E. tancrei *	54	21062	♂	7	Kyrgyzstan. Highway Bishkek - Chatyr-Kel', 270 km, 4 km after Sary-Bulak settlement	41°55'N 75°43'E	1983	–
48	*E.alaicus* × *E.tancrei* hybrid	53	21063	♀	7	Kyrgyzstan. Highway Bishkek - Chatyr-Kel', 270 km, 4 km after Sary-Bulak settlement	41°55'N 75°43'E	1983	–
49	* E. tancrei *	54	21064	♂	7	Kyrgyzstan. Highway Bishkek - Chatyr-Kel', 270 km, 4 km after Sary-Bulak settlement	41°55'N 75°43'E	1983	–
50	* E. tancrei *	54	21065	♀	7	Kyrgyzstan. Highway Bishkek - Chatyr-Kel', 270 km, 4 km after Sary-Bulak settlement	41°55'N 75°43'E	1983	–
51	* E. tancrei *	54	21072	♂	7	Kyrgyzstan. Highway Bishkek - Chatyr-Kel', 270 km, 4 km after Sary-Bulak settlement	41°55'N 75°43'E	1983	–
52	* E. tancrei *	54	21073	♀	7	Kyrgyzstan. Highway Bishkek - Chatyr-Kel', 270 km, 4 km after Sary-Bulak settlement	41°55'N 75°43'E	1983	–
53	* E. tancrei *	54	21074	♂	7	Kyrgyzstan. Highway Bishkek - Chatyr-Kel', 270 km, 4 km after Sary-Bulak settlement	41°55'N 75°43'E	1983	–
54	* E. tancrei *	54	21075	♂	7	Kyrgyzstan. Highway Bishkek - Chatyr-Kel', 270 km, 4 km after Sary-Bulak settlement	41°55'N 75°43'E	1983	–
55	* E. tancrei *	54	21076	♀	7	Kyrgyzstan. Highway Bishkek - Chatyr-Kel', 270 km, 4 km after Sary-Bulak settlement	41°55'N 75°43'E	1983	–
56	* E. tancrei *	54	21077	♀	7	Kyrgyzstan. Highway Bishkek - Chatyr-Kel', 270 km, 4 km after Sary-Bulak settlement	41°55'N 75°43'E	1983	–
57	* E. tancrei *	54	21078	♀	7	Kyrgyzstan. Highway Bishkek - Chatyr-Kel', 270 km, 4 km after Sary-Bulak settlement	41°55'N 75°43'E	1983	–
58	* E. alaicus *	48	25600	♂	8	Tajikistan. The right bank of the Kyzyl-Suu River, 4 km to the East from the Achek-Alma settlement, 2160 m above sea level	39°22.73'N 71°40.68'E	2010	–
59	* E. alaicus *	48	25605	♀	8	Tajikistan. The right bank of the Kyzyl-Suu River, 4 km to the East from the Achek-Alma settlement, 2160 m above sea level	39°22.73'N 71°40.68'E	2010	MG264322
60	* E. alaicus *	48	25610	♀	8	Tajikistan. The right bank of the Kyzyl-Suu River, 4 km to the East from the Achek-Alma settlement, 2160 m above sea level	39°22.73'N 71°40.68'E	2010	MG264323
61	* E. alaicus *	48	25611	♂	8	Tajikistan. The right bank of the Kyzyl-Suu River, 4 km to the East from the Achek-Alma settlement, 2160 m above sea level	39°22.73'N 71°40.68'E	2010	MG264324
62	* E. alaicus *	48	25612	♀	8	Tajikistan. The right bank of the Kyzyl-Suu River, 4 km to the East from the Achek-Alma settlement, 2160 m above sea level	39°22.73'N 71°40.68'E	2010	MG264325
63	* E. alaicus *	48	25622	♀	8	Tajikistan. The right bank of the Kyzyl-Suu River, 4 km to the East from the Achek-Alma settlement, 2160 m above sea level	39°22.73'N 71°40.68'E	2010	–
64	* E. alaicus *	50	20054	♀	8	Tajikistan. The right bank of the Kyzyl-Suu River, 4 km to the East from the Achek-Alma settlement, 2160 m above sea level	39°22.73'N 71°40.68'E	1981	–
65	* E. alaicus *	50–51	20053	♂	8	Tajikistan. The right bank of the Kyzyl-Suu River, 4 km to the East from the Achek-Alma settlement, 2160 m above sea level	39°22.73'N 71°40.68'E	1981	–
66	* E. alaicus *	50	20050	♂	8	Tajikistan. The right bank of the Kyzyl-Suu River, 4 km to the East from the Achek-Alma settlement, 2160 m above sea level	39°22.73'N 71°40.68'E	1981	–
67	* E. alaicus *	48	25602	♀	9	Tajikistan. The left bank of the Kyzyl-Suu River, in front of the Duvana settlement, 2000 m above sea level	39°20.7'N 71°34.73'E	2010	MG264326
68	* E. alaicus *	48	27023	♀	9'	Tajikistan. The left bank of the Kyzyl-Suu River, in front of the Duvana settlement, 2000 m above sea level	39°20.588'N 71°34.528'E	2018	–
69	* E. alaicus *	48	27024	♂	9'	Tajikistan. The left bank of the Kyzyl-Suu River, in front of the Duvana settlement, 2000 m above sea level	39°20.588'N 71°34.528'E	2018	–
70	* E. alaicus *	48	27025	♂	10	Tajikistan. The left bank of the Kyzyl-Suu River, close to Dzhailgan settlement	39°19.277'N 71°32.772'E	2018	MK544910
71	* E. alaicus *	48	27026	♂	10	Tajikistan. The left bank of the Kyzyl-Suu River, close to Dzhailgan settlement	39°19.277'N 71°32.772'E	2018	MK544911
72	* E. alaicus *	48	27028	♀	11	Tajikistan. The left bank of the Kyzyl-Suu River, 3 km to the East from the bridge to Kashat settlement	39°18.449'N 71°28.480'E	2018	MK544913
73	* E. alaicus *	48	27029	♀	11	Tajikistan. The left bank of the Kyzyl-Suu River, 3 km to the East from the bridge to Kashat settlement	39°18.449'N 71°28.480'E	2018	MK544914
74	* E. alaicus *	48	27030	♀	12	Tajikistan. The left bank of the Muksu River, close to Sary-Tala settlement	39°14.748'N 71°25.000'E	2018	MK544915
75	* E. alaicus *	48	27031	♀	12	Tajikistan. The left bank of the Muksu River, close to Sary-Tala settlement	39°14.748'N 71°25.000'E	2018	–
76	* E. alaicus *	48	27032	♀	12	Tajikistan. The left bank of the Muksu River, close to Sary-Tala settlement	39°14.748'N 71°25.000'E	2018	MK544916
77	* E. alaicus *	48	27033	♂	12	Tajikistan. The left bank of the Muksu River, close to Sary-Tala settlement	39°14.748'N 71°25.000'E	2018	MK544917
78	* E. tancrei *	54	27019	♂	13	Tajikistan. Pamir-Alay, close to Utol Poyon settlement, the southern bank of the Surkhob River	39°9.737'N 71°7.374'E	2018	MK544906
79	* E. tancrei *	54	27020	♀	13	Tajikistan. Pamir-Alay, close to Utol Poyon settlement, the southern bank of the Surkhob River	39°9.737'N 71°7.374'E	2018	MK544907
80	* E. tancrei *	54	27021	♂	13	Tajikistan. Pamir-Alay, close to Utol Poyon settlement, the southern bank of the Surkhob River	39°9.737'N 71°7.374'E	2018	MK544908
81	* E. tancrei *	54	27022	♀	13	Tajikistan. Pamir-Alay, close to Utol Poyon settlement, the southern bank of the Surkhob River	39°9.737'N 71°7.374'E	2018	MK544909
82	* E. tancrei *	54	27017	♂	14	Tajikistan. Pamir-Alay, between settlements Kichikzy – Utol Poyon, the southern bank of the Surkhob River	39°7.625'N 70°59.762'E	2018	MK544904
83	* E. tancrei *	54	27018	♀	14	Tajikistan. Pamir-Alay, between settlements Kichikzy – Utol Poyon, the southern bank of the Surkhob River	39°7.625'N 70°59.762'E	2018	MK544905
84	* E. tancrei *	54	27027	♂	14	Tajikistan. Pamir-Alay, between settlements Kichikzy – Utol Poyon, the southern bank of the Surkhob River	39°7.625'N 70°59.762'E	2018	MK544912
85	* E. tancrei *	52	24898	♂	15	Tajikistan. Pamir-Alay, close to Kichikzy settlement, the southern bank of the Surkhob River	39°8.23'N 70°57.33'E	2008	MK544900
86	* E. tancrei *	51	24899	♀	15	Tajikistan. Pamir-Alay, close to Kichikzy settlement, the southern bank of the Surkhob River	39°8.23'N 70°57.33'E	2008	
87	* E. tancrei *	30	25601	♀	16	Tajikistan. Pamir-Alay, close to the settlement Shilbili, the northern bank of the Surkhob River, 1900 m above sea level	39°15.37'N, 71°20.59'E	2010	MG264327
88	* E. tancrei *	30	25618	♀	16	Tajikistan. Pamir-Alay, close to the settlement Shilbili, the northern bank of the Surkhob River, 1900 m above sea level	39°15.37'N 71°20.59'E	2010	MG264328
89	* E. tancrei *	30	25625	♂	16	Tajikistan. Pamir-Alay, close to the settlement Shilbili, the northern bank of the Surkhob River, 1900 m above sea level	39°15.37'N 71°20.59'E	2010	MG264329
90	* E. tancrei *	30	25626	♀	16	Tajikistan. Pamir-Alay, close to the settlement Shilbili, the northern bank of the Surkhob River, 1900 m above sea level	39°15.37'N 71°20.59'E	2010	MG264330
91	* E. tancrei *	48	24872	♀	17	Tajikistan. Pamir-Alay, the right bank of the Surkhob River, close to the airport Garm, 1310 m above sea level	39°0.28'N 70°17.77'E	2008	MG264331
92	* E. tancrei *	48	24873	♀	17	Tajikistan. Pamir-Alay, the right bank of the Surkhob River, close to the airport Garm, 1310 m above sea level	39°0.28'N 70°17.77'E	2008	MG264332
93	* E. tancrei *	48	24874	♂	17	Tajikistan. Pamir-Alay, the right bank of the Surkhob River, close to the airport Garm, 1310 m above sea level	39°0.28'N 70°17.77'E	2008	MG264333
94	* E. tancrei *	48	24876	♂	17	Tajikistan. Pamir-Alay, the right bank of the Surkhob River, close to the airport Garm, 1310 m above sea level	39°0.28'N 70°17.77'E	2008	MG264334
95	* E. tancrei *	48	24914	♀	17	Tajikistan. Pamir-Alay, the right bank of the Surkhob River, close to the airport Garm, 1310 m above sea level	39°0.28'N 70°17.77'E	2008	MG264335
96	* E. tancrei *	48	24915	♂	17	Tajikistan. Pamir-Alay, the right bank of the Surkhob River, close to the airport Garm, 1310 m above sea level	39°0.28'N 70°17.77'E	2008	MG264336
97	* E. tancrei *	50	24904	♀	18	Tajikistan. Pamir-Alay, the left bank of the Surkhob River near the Shulonak, on the way to Voidara settlement, 1300 m above sea level	38°59.3'N 70°16.1'E	2008	MG264337
98	* E. tancrei *	50	24911	♂	19	Tajikistan. Pamir-Alay, the left bank of the Surkhob River near the Voydara settlement, 1440 m above sea level	38°58.9'N 70°14.71'E	2008	–
99	* E. tancrei *	50	24907	♀	19	Tajikistan. Pamir-Alay, the left bank of the Surkhob River near the Voydara settlement, 1440 m above sea level	38°58.9'N 70°14.71'E	2008	MG264338
100	* E. tancrei *	50	24910	♂	19	Tajikistan. Pamir-Alay, the left bank of the Surkhob River near the Voydara settlement, 1440 m above sea level	38°58.9'N 70°14.71'E	2008	MG264339
101	* E. tancrei *	54	20769	♂	20	Uzbekistan. Close to Sokh settlement, 11 km to the west	39°58'N 70°58'E	1983	–
102	* E. tancrei *	54	20770	♀	20	Uzbekistan. Close to Sokh settlement, 11 km to the west	39°58'N 70°58'E	1983	–
103	* E. tancrei *	54	20772	♂	20	Uzbekistan. Close to Sokh settlement, 11 km to the west	39°58'N 70°58'E	1983	–
104	* E. tancrei *	54	20773	♀	20	Uzbekistan. Close to Sokh settlement, 11 km to the west	39°58'N 70°58'E	1983	–
105	* E. tancrei *	54	25159	♂	21	Uzbekistan. Tashkent city	41°20.49'N 70°18.71'E	2009	MG264346
106	* E. tancrei *	54	20561	♀	22	Kyrgyzstan. The Southern bank of the Issyk-Kel' Lake, 16 km to the South from the Barskaun settlement, Lake Barskaun canyon	42°00'N 77°37'E	1982	–
107	* E. tancrei *	54	20562	♂	22	Kyrgyzstan. The Southern bank of the Issyk-Kel' Lake, 16 km to the South from the Barskaun settlement, Lake Barskaun canyon	42°00'N 77°37'E	1982	–
108	* E. tancrei *	54	24912	♂	23	Tajikistan. The northern bank of the Vakhsh River, Miskinobod, 1780 m above sea level	38°39.78'N 69°33.29'E	2008	MG264344
109	* E. tancrei *	54	24913	♂	24	Tajikistan. Panchkotan gorge, left bank of the Sorbo River, close to Romit reserve, 1265 m above sea level	38°45.27'N 69°17.6'E	2008	MG264345
110	* E. tancrei *	50	24905	♂	25	Tajikistan. The Varzob Valley, near the Khodzha-Obi-Garm settlement, 2000 m above sea level	38°53.53'N 68°46.52'E	2008	MG264340
111	* E. tancrei *	50	24906	♂	25	Tajikistan. the Varzob Valley, near the Khodzha-Obi-Garm settlement, 2000 m above sea level	38°53.53'N 68°46.52'E	2008	MG264341
112	* E. tancrei *	50	24916	♀	25	Tajikistan. the Varzob Valley, near the Khodzha-Obi-Garm settlement, 2000 m above sea level	38°53.53'N 68°46.52'E	2008	MG264342
113	* E. tancrei *	50	24917	♂	25	Tajikistan. the Varzob Valley, near the Khodzha-Obi-Garm settlement, 2000 m above sea level	38°53.53'N 68°46.52'E	2008	MG264343
114	* E. tancrei *	54	27016	♂	26	Tajikistan. Khatlon district, close to Sovetabad settlement	37°28.479'N 68°15.568'E	2018	MK544903
115	* E. tancrei *	54	27013	♂	27	Tajikistan. Khatlon district, close to Aivadj settlement	36°58.168'N 68°0.791'E	2018	MK544901
116	* E. tancrei *	54	27014	♂	27	Tajikistan. Khatlon district, close to Aivadj settlement	36°58.168'N 68°0.791'E	2018	MK544902
117	* E. talpinus *	54	24736	♀	28	Russia. Orenburg oblast, Belyaevsky district, about 15 km southeast of the Belyaevka village	51°14'N 56°38'E	2005	MG264347
118	* E. talpinus *	54	26910	♂	29	Russia. Samara oblast, Stavropolsky rayon, Samarskaya Luka	53°9.98'N 49°35.35'E	2016	MG264354
119	* E. talpinus *	–	26491	♀	30	Russia. Crimea, Bakhchisaraysky district, 2 km south of the Sevastyanovka village	44°47.82'N 33°55.95'E	2013	MG264359
120	* E. talpinus *	–	26493	♀	30	Russia. Crimea, Bakhchisaraysky district, 2 km south of the Sevastyanovka village	44°47.82'N 33°55.95'E	2013	*cytb* mitotype is identical to MG264359
121	* E. talpinus *	54	26800	♀	31	Russia. Omsk oblast, Tavrichesky district, near the Novouralsky railway station, about 16 km south-east of the Novouralsky village	54°14.586'N 74°17.66'E	2014	MG264351
122	* E. talpinus *	54	26802	♀	32	Russia. Novosibirsk oblast, Tatarsky district, near the Novopervomayskoe village and Lagunaka railway station	55°8.64'N 75°21.94'E	2014	MG264352
123	* E. talpinus *	54	26850	♂	33	Russia. Omsk oblast, Cherlaksky district, approximately 3.5 km northeast of the Irtysh village	54°30.59'N 74°25.95'E	2015	MG264353

### Samples

We used samples from the Joint collection of wildlife tissues for fundamental, applied and environmental researches of the Koltzov Institute of Developmental Biology RAS, the state registration number AAAA-A16-116120810085-1, which is a part of the Core Centrum of the Koltzov Institute of Developmental Biology RAS, the state registration number 6868145. Tissues and chromosome suspensions were collected during our field trips in 1981–1983, 2008, 2010, 2013, and 2015–2018. For *cytb* sequencing we also used dried skins of specimens S132130*, S132131*, S132133*, S132135* deposited to the Zoological Museum of Lomonosov Moscow State University (Table 1) and originated from the *terra typica* of the Alay mole vole.

Animals were treated according to established international protocols, as in the Guidelines for Humane Endpoints for Animals Used in Biomedical Research. All the experimental protocols were approved by the Ethics Committees for Animal Research of the Koltzov Institute of Developmental Biology RAS in accordance with the Regulations for Laboratory Practice in the Russian Federation. All efforts were made to minimize animal suffering.

### Mitotic and meiotic chromosomes

Chromosomes from bone marrow were prepared according to Ford and Hamerton (1956) for all animals listed with chromosome numbers in Table 1. G-banding was achieved using trypsin digestion (Seabright 1971). Samples from 3 animals (25610, 25611, 25612, Table 1) were used for tissue culture (Stanyon and Galleni 1991, Romanenko et al. 2015). All cell lines were retrieved from the IMCB SB RAS cell bank (“The general collection of cell cultures”, № 0310-2016-0002). Full sets of paints derived from flow-sorted chromosomes of the field vole *Microtusagrestis* Linnaeus, 1761 were used (Sitnikova et al. 2007). FISH was performed according to previously published protocols (Yang et al. 1999, Graphodatsky et al. 2000). G-banding was carried out for metaphase chromosomes prior to FISH. The same procedures were used previously for specimens from localities 11, 12, 16, 17, and 18 (Bakloushinskaya et al. 2010, 2012, 2013, Matveevsky et al. 2015). It was not possible to use Zoo-FISH on material gathered in the 1980s, but the pictures of G-banded karyotypes were suitable for comparative analyses. Karyological data, obtained from 1981 to 2008, were re-examined in accordance with a new nomenclature for the Rb translocations in *E.tancrei* (Bakloushinskaya et al. 2012, 2013). In total, we studied chromosomes for 114 specimens of *E.alaicus*, *E.tancrei* and *E.talpinus*.

Images were captured using VideoTesT-FISH 2.0. and VideoTesT-Karyo 3.1. (Imicrotec) or Case Data Manager 6.0 (Applied Spectral Imaging Inc., ASI) software with either ProgRes CCD (Jenoptik) or ASI CCD camera, respectively, mounted on an Axioskop 2 plus (Zeiss) microscope with filter sets for DAPI, FITC, and rhodamine. Hybridization signals were assigned to specific chromosome regions defined by GTG-banding patterns previously captured with the CCD camera. Routine and G-banded plates were captured with a CMOS camera, mounted on an Axioskop 40 (Zeiss) microscope. Images were processed using Paint Shop Pro X2 (Corel).

The suspensions and spreads of spermatocytes of two *E.alaicus* males (27024, 27025) were made as described by Kolomiets et al. (2010). Immunostaining was designed as in our previous studies (Kolomiets et al. 2010, Matveevsky et al. 2016). Synaptonemal complexes (SC) and centromeres in pachytene spermatocytes were detected using antibodies to axial SC elements – SYCP3 (Abcam, UK) and the kinetochores (CREST, Fitzgerald Industries International, USA). The slides were analyzed with an Axioimager D1 microscope (Carl Zeiss, Jena, Germany). Images were processed using Adobe Photoshop CS3 Extended.

### *cytb* sequencing

Total DNA was isolated by phenol-chloroform deproteinisation after treatment of shredded tissues with proteinase K (Sambrook et al. 1989). The primers used for amplification and sequencing of the complete *cytb* gene (1143 bp) in species of the Ellobius subgenus are listed in Table 2. Polymerase chain reaction (PCR) was carried out in a mixture containing 25–50 ng DNA, 2 µl 10×Taq-buffer, 1.6 µl 2.5 mM dNTP solution, 4 pM of each primer, 1 unit of Taq-polymerase, and deionized water to a final volume of 20 µL. Amplification was as follows: preheating at 94 °C for 3 min, then 35 cycles in a sequential mode of 30 s at 94 °C, 1 min at 55 or 57 °C depending on the applied pair of primers, and 1 min at 72 °C; the reaction was completed by a single final elongation of PCR products at 72 °C for 6 min. Automatic sequencing was carried out using a PRISM BigDye TM Terminator v. 3.1 kit (ABI, United States) with ABI 3500 genetic analyzer at the Core Centrum of the Koltzov Institute of Developmental Biology RAS.

**Table 2.** Primers, which were used for amplification and sequencing of *cytb* gene in mole voles of the Ellobius subgenus. Primers Eta_CytbF1, and VOLE14 were used to amplify the full *cytb* gene with flanked fragments of mtDNA; all other primers correspond to various internal areas of *cytb* gene, the position of their 5’-end nucleotide from the start of *cytb* gene is in parentheses.

Species	Primer designation	Nucleotide sequence of primer (5’–3’) and its localization within the full gene *cytb*	Citation
* E. talpinus *	Forward primers		
	Eta_CytbF1	GAAACACCTAATGACAATCATACG	Bogdanov et al. 2015
	L15095-Ell	(370)-ATAGCCACAGCATTCATA	Bogdanov et al. 2015
	L15473-Ell	(748)-CTCGGAGACCCAGATAACTAC	Bogdanov et al. 2015
	Reverse primers		
	MVZ04m	(431)-GTGGCCCCTCAAAATGATATTTGTCCTC	Bogdanov et al. 2015
	CLETH16m	(824)-AGGAAGTACCATTCTGGTTTAAT	Bogdanov et al. 2015
	VOLE14	TTTCATTACTGGTTTACAAGAC	Conroy and Cook 1999
*E.tancrei*, *E.alaicus*	Forward primers		
	Eta_CytbF1	GAAACACCTAATGACAATCATACG	Bogdanov et al. 2015
	L15095-Ell	(370)-ATAGCCACAGCATTCATA	Bogdanov et al. 2015
	Vole23m	(590)-TCCTGTTCCTTCACGAAACAGGTTC	Bogdanov et al. 2015
	L15473-Elal	(748)-CTTGGAGACCCAGACAATTTC	Our design
	Reverse primers		
	MVZ04m	(431)-GTGGCCCCTCAAAATGATATTTGTCCTC	Bogdanov et al. 2015
	CLETH16m	(824)-AGGAAGTACCATTCTGGTTTAAT	Bogdanov et al. 2015
	VOLE14	TTTCATTACTGGTTTACAAGAC	Conroy, Cook 1999

A total of 53 samples of the subgenus Ellobius mole voles were used for mitochondrial *cytb* gene sequencing; all sequences have been deposited in GenBank, accession numbers MG264318–MG264347, MG264351–MG264354, MG264359, and MK544900- MK544917 (http://www.ncbi.nlm.nih.gov/genbank/) are listed in the Table 1.

## Results

### Karyotyping

**Figure 2.**
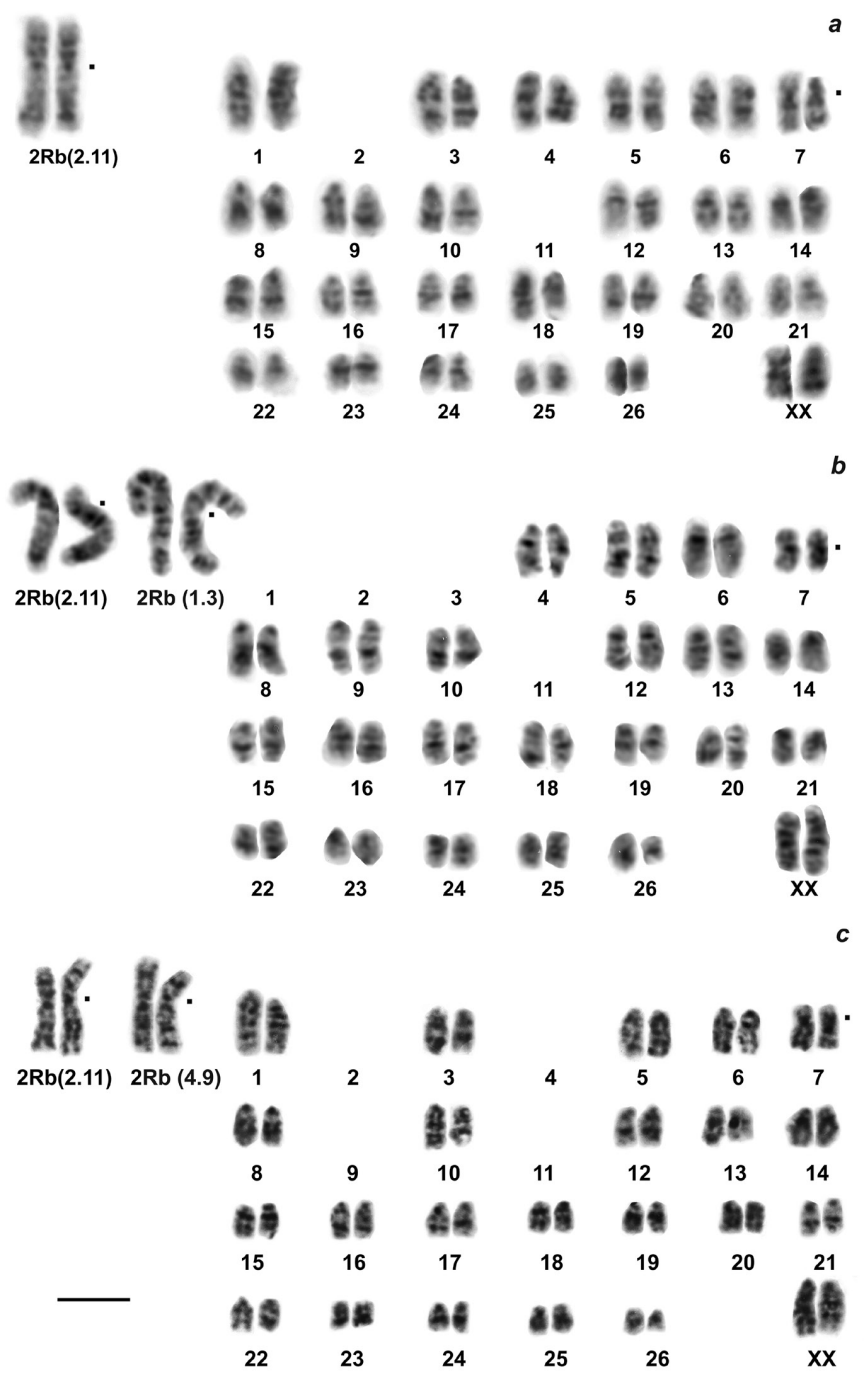
G-banded karyotypes of *E.alaicus***a** 2n = 52, 21071, ♂, locality #6 **b** 2n = 50, 21089, ♂, locality #6 **c** 2n = 50 20054, ♀, locality #8. The chromosome nomenclature follows Bakloushinskaya et al. (2012, 2013). Black dots mark the positions of centromeres in bi-armed chromosomes. Scale bar: 10 μm.

**Figure 3.**
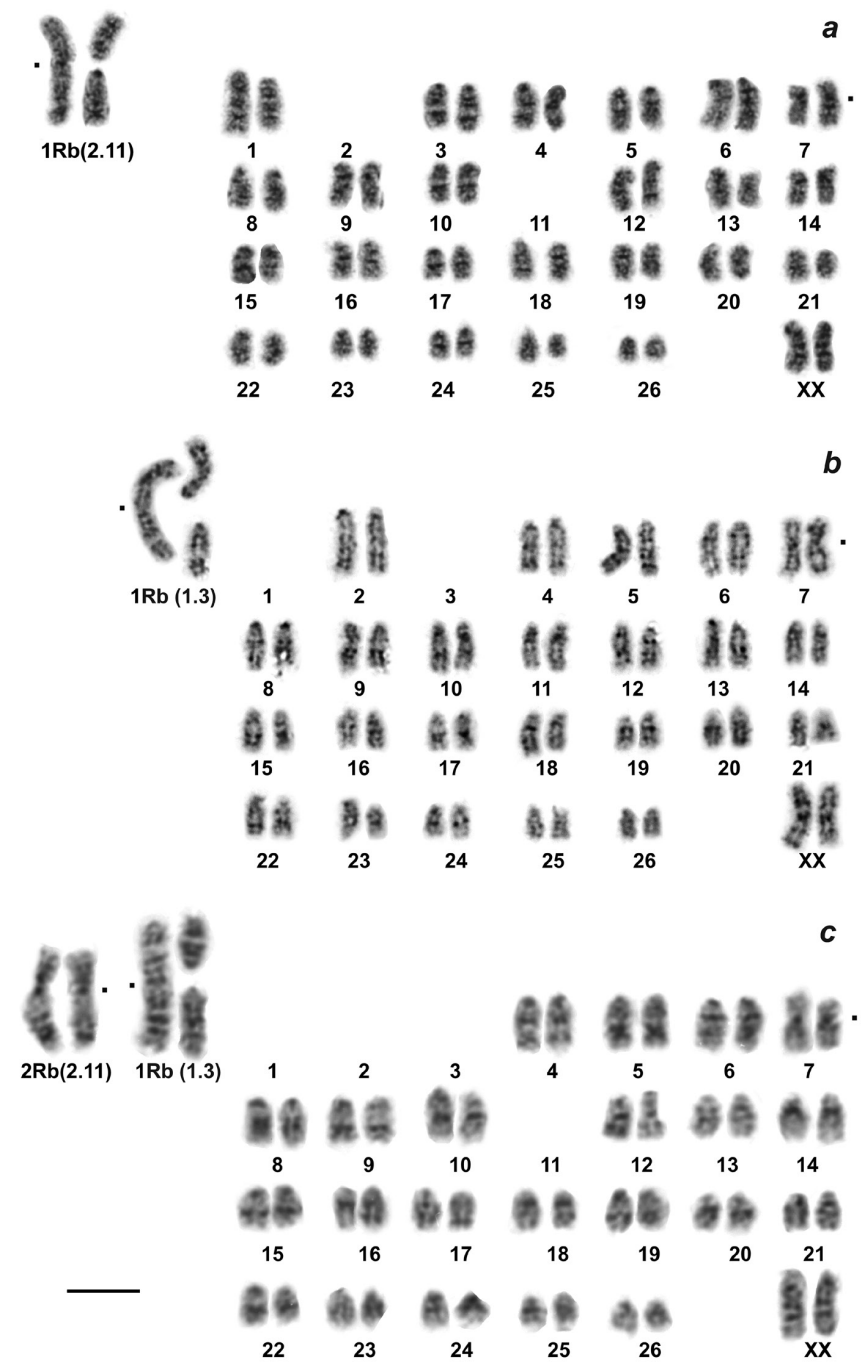
G-banded karyotypes of heterozygous mole voles **a** 2n = 53 20778, ♂, locality #3 **b** 2n = 53, 21059, ♀, locality #7 **c** 2n = 51, 21070, ♀, locality #6. Scale bar: 10 μm.

The main result was a discovery of specific chromosome variability in *E.alaicus*, with 2n varying from 52 to 48 chromosomes. For mole voles from the Alay Ridge, the Naryn Valley, and the Aksai River Valley (localities # 3, 5, 6, Fig. 1, Table 1) we described 2n = 52 with two homozygous Robertsonian translocations, which was counted as 2.11 [2 Rb(2.11)] according to *E.tancrei* chromosome nomenclature ([Bibr B7][Bibr B3]) (Fig. 2a). The northern side of the Alay Ridge slopes down to the Ferghana Valley, where *E.tancrei*, 2n = 54, exists (# 20). Hybrids with 2n = 53, heterozygous by the same translocation [1 Rb(2.11)] (Fig. 3a), were found at the northern slopes of the Alay Ridge (# 3), which marks the species contact zone. The Ferghana Ridge separates the Alay Valley from the Chatyr-Kel’ Lake Basin, the Aksai River Valley, and the Naryn Valley, one of the largest within the Inner Tien Shan. Fascinating data were obtained for animals inhabiting the Chatyr-Kel’ Lake surrounds and the Naryn district (localities # 4 and 6, Table 1, Fig. 1), where we found Alay mole voles with 2n = 50, and heterozygotes with 2n = 51, which are presumed hybrids with typical *E.alaicus*, 2n = 52 (Figs 2b, 3c). Chromosomal number in animals with 2n = 50 was decreased because of another translocation, the Rb(1.3). Nevertheless, in the Aksai River Valley, the typical *E.alaicus* with 2n = 52 [2 Rb(2.11)] were found (locality # 5, Table 1, Fig. 1).

Two heterozygous karyotypes with 2n = 53 due to the presence of different Rb metacentrics were found. In point # 3, we found animals with 2n = 53 and 1 Rb(2.11), which are hybrids of *E.alaicus* and *E.tancrei* (Fig. 3a). Mole voles with 2n = 53 from the Naryn district (#7, Fig. 1, Table 1) had another translocation, 1 Rb(1.3) (Fig. 3b). We were not able to find animals with 2n = 52 and 2 Rb(1.3), but probably they inhabit an extensive unstudied area in the Naryn Valley, between points #6 and 7.

**Figure 4.**
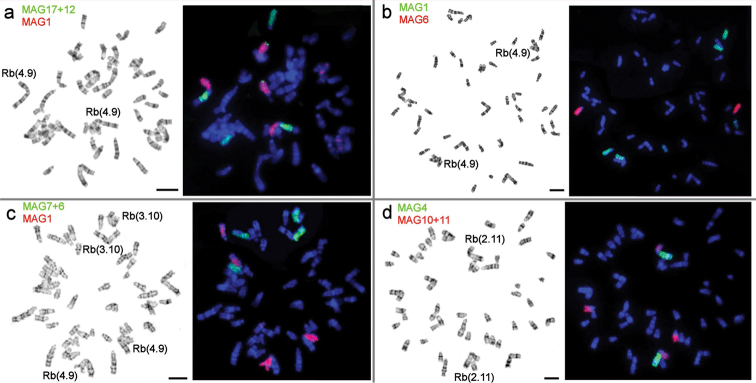
Fluorescent *in situ* hybridization of *M.agrestis* (MAG) probes on *E.alaicus* metaphase chromosomes, 2n = 48 (locality #8): **a** MAG 1 (red) and MAG 17+12 (green), 25610 ♀, locality #8; **b** MAG 1 (green) and MAG 6 (red), 25610 ♀, locality #8; **c** MAG 1 (red) and MAG 7+6 (green), 25612 ♀, locality #8; **d** MAG 4 (green) and MAG 10+11 (red), 25612 ♀, locality #8. Scale bar: 10 μm.

**Figure 5.**
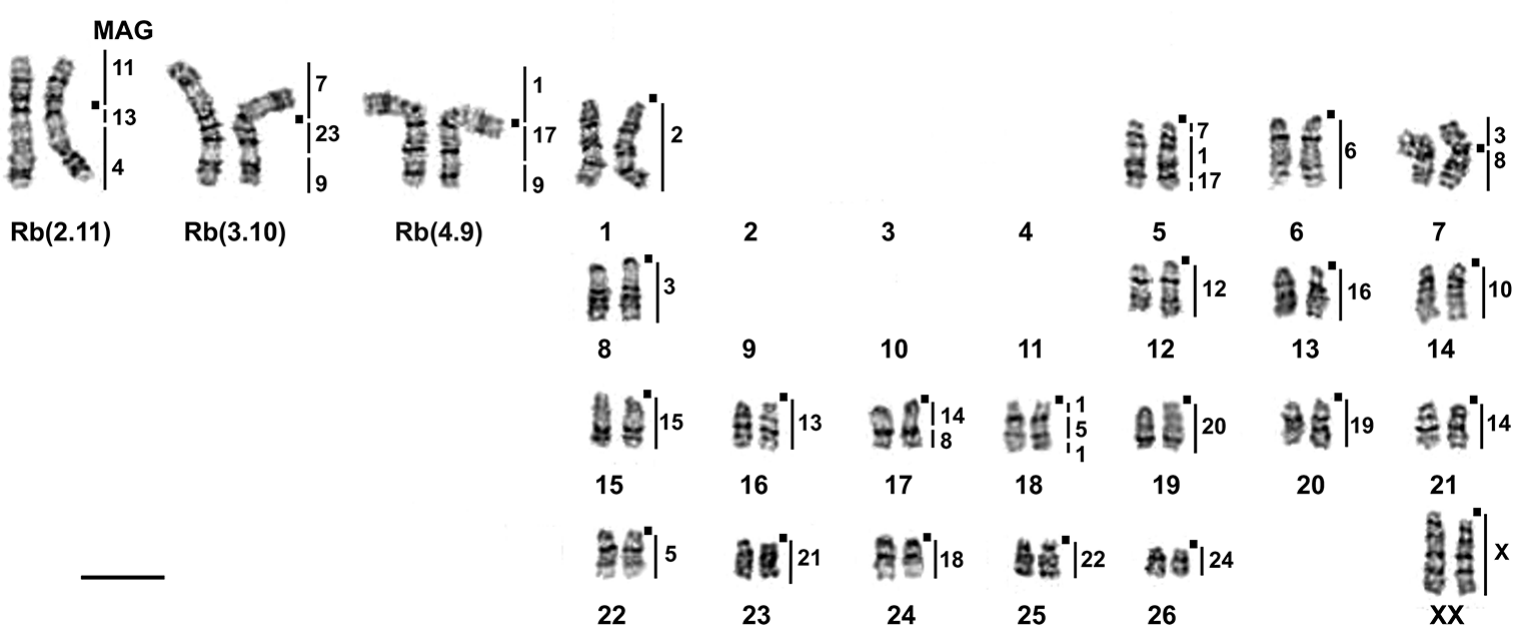
G-banded karyotype of a new form of *E.alaicus*, 2n = 48, 2 Rb (2.11), 2 Rb (4.9), 2 Rb (3.10), 25610 ♀, locality #8. The chromosome nomenclature follows Bakloushinskaya et al. (2012, 2013). Black squares mark the positions of centromeres. Vertical black bars and the numbers beside them mark the localization of *M.agrestis* (MAG) chromosome segments. Scale bar: 10 μm.

The most surprising data we revealed for animals from the Pamir-Alay mountains, Tajikistan, (# 8, Fig. 1, Table 1). In 1981, we got Alay mole voles from there for breeding and karyotyping; two animals had 2n = 50, and one was a somatic mosaic, 2n = 50-51. Their karyotypes included 2 Rb(2.11) and 1–2 Rb(4.9); the last one was heterozygous in the mosaic specimen (Fig. 2c). After almost 30 years (in 2010) we caught animals with 2n = 48 at the same locality, and one mole vole with the same karyotype at the opposite bank of the Kyzyl-Suu River (locality # 9, Fig. 1, Table 1). Their karyotypes contained one more pair of Rb metacentrics, Rb(3.10). The entire set of Rbs was 2 Rb(2.11), 2 Rb(4.9), 2 Rb(3.10), all of which were confirmed by chromosome painting for specimens 25610, 25611, 25612 (Figs 4, 5). The 21 MAG (*Microtusagrestis*) autosomal probes revealed 35 conserved segments in the mole voles’ genome, which corresponds to the genome composition of the typical *E.tancrei*, 2n = 54 (Bakloushinskaya et al. 2012), and its form with the lowest chromosome number, 2n = 30 (Bakloushinskaya et al. 2013). The MAG X chromosome probe produced signals on both male and female X chromosomes; the MAG Y probes did not demonstrate any specific signal. Therefore, we suppose that *E.alaicus* has the same isomorphic sex chromosomes, XX in both sexes, as *E.talpinus* and *E.tancrei*.

In 2018 we checked chromosome sets for Alay mole voles from the Kyzyl-Suu River Valley, the Kyzyl-Suu and Muksu Rivers interfluve, and the left bank of the Muksu River (localities # 9–12, Fig. 1, Table 1). All 10 studied animals have 2n = 48 [2 Rb(2.11), 2 Rb(4.9), 2 Rb(3.10)].

In total we described seven variants of karyotypes for *E.alaicus* (Table 1, Figs 2, 3, 5): 2n = 48, 50 (two forms), 51, 52, 53 (two variants) with four different Rb translocations Rb(2.11), Rb(1.3), Rb(4.9), Rb(3.10) in different combinations. We assumed, by comparing our data on G-banded karyotypes and chromosomal painting, that the Rb(2.11) is typical for *E.alaicus*. This translocation was revealed in all specimens of the species (Table 1, Figs 2, 3a,c), excluding interspecific hybrids of *E.tancrei* and *E.alaicus* from the Naryn district 2n = 53, 1 Rb(1.3) (Table 1, Fig. 3b), see Discussion.

## Discussion

A few studies dealt with *Ellobius* molecular phylogeny before. Conroy and Cook (1999) studied *cytb* of two species, *E.fuscocapillus* and *E.tancrei*, and their position in the Arvicolinae tree appeared to be unstable in different models. Data on variations of short fragments of nuclear genes (partial *LCAT* and exon 10 *GHR*) in *E.talpinus* and *E.tancrei* contradicted the conventional view that *Ellobius* is an ancient group because of simplicity of rooted molars and the peculiar structure of the skull (Abramson et al. 2009). Fabre et al. (2012) re-analyzed these data among others for comparative meta-analyses of the rodent diversity and phylogeny without special attention to Ellobiini. Nevertheless, the genus *Ellobius* appears to be a young group; its morphological characters indicate adaptation to subterranean life and provide no phylogenetic signal. *E.talpinus* and *E.tancrei* separated not earlier than the latest Pliocene and Early Pleistocene between ca. 2.1–1.0 Ma (Abramson et al. 2009). The phyletic lineage leading to the recent *E.talpinus* includes at least two chronospecies: Late Pliocene-Early Pleistocene *E.kujalnikensis* and early Middle Pleistocene *E.melitopoliensis*; *E.talpinus* was recognized from the late Middle Pleistocene (Tesakov 2009). There are no such data for *E.tancrei* and *E.alaicus*.

Here, for the first time, we demonstrated data on molecular, mitochondrial (*cytb*) and nuclear (*XIST* and *Rspo1* fragments) specificity of *E.alaicus*. The *cytb* variability in the subgenus Ellobius, which we demonstrated here, is comparable and even higher than in *Ctenomys*, subterranean rodents with numerous species-specific chromosome changes (Buschiazzo et al. 2018). In *Ctenomys* genetic distances, calculated on *cytb* gene, range from 0 to 2.28%, whereas 2n varies from 41 to 70, and autosomal fundamental numbers (NFa) from 72 to 84. Nevertheless, *cytb* appears to be more informative for phylogenetic reconstructions compared to nuclear markers. Published data on partial sequences of *XIST* and *Sox9* revealed no differences for *E.talpinus* and *E.tancrei* (Just et al. 2007, Bagheri-Fam et al. 2012). Our data on fragments of *Eif2s3x* and *Eif2s3y* for *E.talpinus*, *E.tancrei*, and *E.alaicus* also reveal no changes in the exonic part of the genes (Matveevsky et al. 2017). The cryptic *Ellobius* species are rather young ones, so this may be why nuclear DNA markers were insufficient. However, our new data on *XIST* and *Rspo1* variability demonstrated apparent clustering for all species of the Ellobius subgenus despite interspecific genetic distances were rather low and relatively high difference of *E.tancrei* specimens from Tajikistan and Uzbekistan, as nuclear markers of the latest (specimen 25159) could not be assigned to any of the two clades.

**Figure 6.**
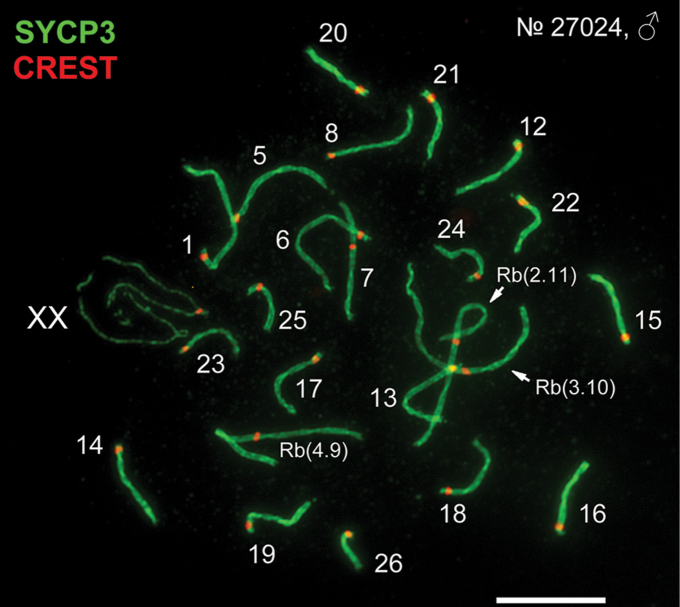
Chromosome synapsis in pachytene spermatocytes of *E.alaicus*, 27024, ♂ (2n = 48, NF = 56), locality #9’. Axial SC elements were identified using anti-SYCP3 antibodies (green), anti-CREST for kinetochores (red). Numbers of SC correspond to chromosome numbers in the karyotype (see Fig. 5). Scale bar: 10 μm.

**Figure 7.**
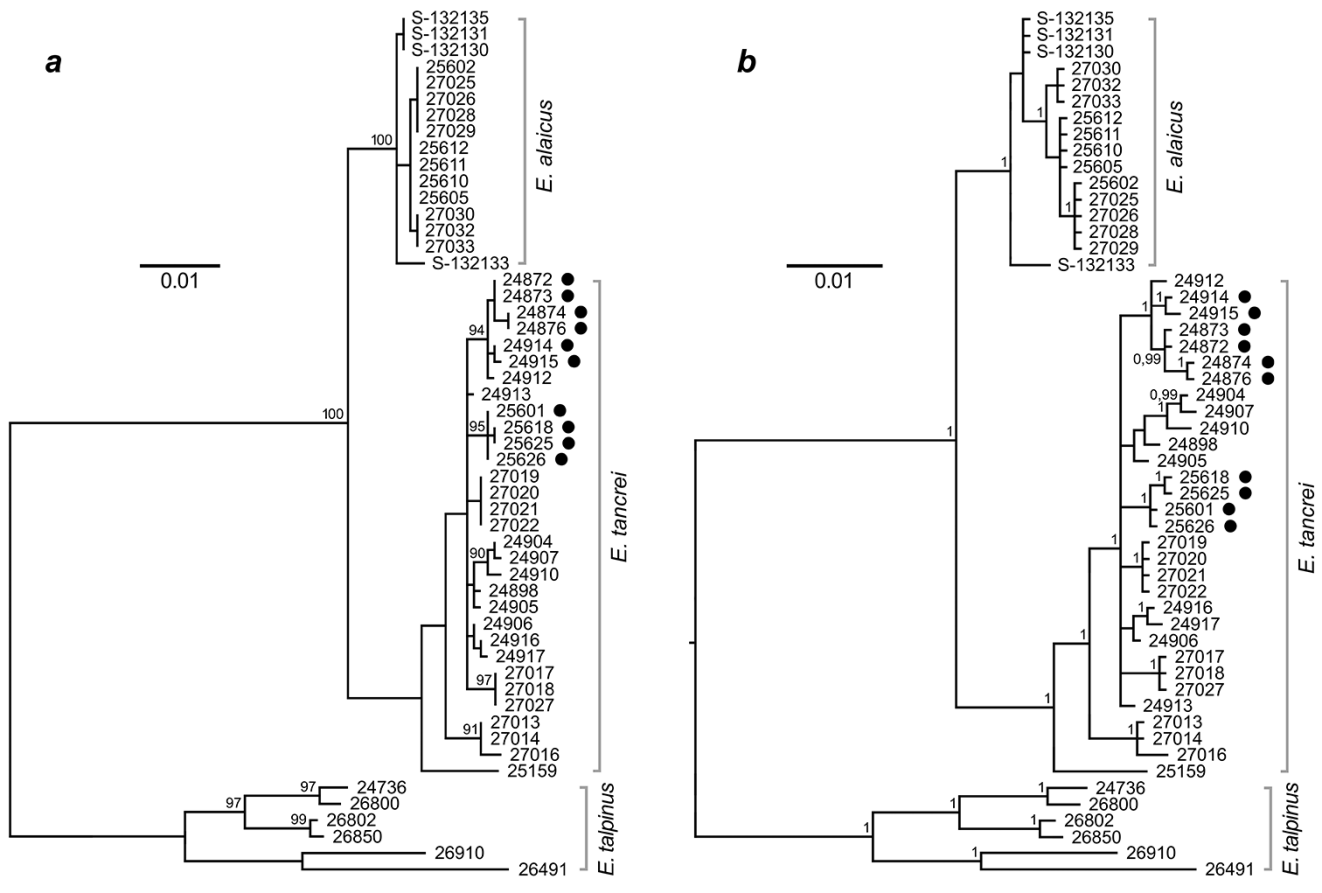
Trees of the subgenus Ellobius inferred from complete mitochondrial *cytb* gene sequences (1143 bp) of 53 specimens **a** a tree was got by using the Maximum Likelihood method based on the Tamura-Nei model, bootstrap support is listed above main branches. Only values greater than 70 percent are shown **b** Bayesian inference tree was made in MrBayes ver. 3.2 (Ronquist et al. 2012), posterior probabilities >0.75 are given above nodes. *E.tancrei* with 2Rb(2.11) were marked by black spots in both trees.

Originally, *E.alaicus* was described as a species with specific karyotype structure, including a pair of very large bi-armed chromosomes (Vorontsov et al. 1969, Lyapunova, Vorontsov 1978). Now we proved, that this Rb(2.11) metacentric is the same as in the *E.tancrei* forms with 2n = 30 and 2n = 48 from the northern bank of the Surkhob River ([Bibr B8][Bibr B3]), but not the Rb(2.18) as in the form with 2n = 50 from the opposite bank of the river. Moreover, translocations Rb(1.3), Rb(4.9), and Rb(3.10) were revealed in the Alay mole voles only. Thus, the Alay mole vole generated a distinctive Robertsonian variability with special structure that highlights genetic distinctness of this species compared to *E.tancrei*. No specimens with 2n = 52 and a single pair of Rb(2.11) were found among over 400 studied *E.tancrei* with Rb translocations (Bakloushinskaya, Lyapunova 2003). Probably, the translocation Rb(2.11) originated independently in *E.alaicus* and *E.tancrei*. The results of the phylogenetic analyses support this assumption because both ML and BI trees demonstrated distant positions for *E.alaicus* and *E.tancrei* specimens carrying Rb(2.11). Their relationships were established indirectly through Uzbekistan and South-West Tajikistan populations of *E.tancrei*, which have no any Robertsonian translocations (Fig. 7).

**Table 3.** Primers, which were used for amplification and sequencing of *XIST* and *Rspo1* genes in the mole voles of the *Ellobius* subgenus.

Nuclear gene	Primer designation	Nucleotide sequence of primer (5’–3’)	Source
*XIST*	Xist1-L11841	GGGGTCTCTGGGAACATTTT	Our design
	Xist1-R12504 or Xist1-Rint	TGCAATAACTCACAAAACCAAC AAGCAGGTAAGTATCCACAGC	Our design
*Rspo1*	Primers used for first amplification		
	Rspo1F-Ell	CACTGTACACTTCCGGGTCTCTTT	Our design
	Rspo1R-Ell	AGAAGTCAACGGCTGCCTCAAGTG	Our design
	Primers used for second PCR with a PRISM®BigDye TM Terminator v. 3.1 kit		
	Rspo1-5intF-Ell	CAGGCACGCACACTAGGTTGTAA	Our design
	Rspo1-1intR-Ell	GTCTAGACTCCCAACACCTG	Our design

Earlier (Lyapunova et al. 1990) we obtained the experimental hybrids of *E.alaicus*, 2n = 52, 2 Rb(2.11) (#3, Fig. 1, Table 1) and *E.tancrei* with 2n = 50, 2 Rb(2.18), 2 Rb(5.9) from the left bank of the Surkhob River (#18, Fig. 1, Table 1). In meiosis, during pachytene I, chains of chromosomes were described (Lyapunova et al. 1990). Now we can explain the results by the partial, monobrachial homology of Rbs involved in the meiotic chains: Rb(2.11) of *E.alaicus* and Rb(2.18) of *E.tancrei*, 2n = 50. Complex chains in meiotic prophase I led to the reduction of fertility in hybrids or even sterility. It might be a possible post-copulation mechanism for reproductive isolation. Here, we demonstrated, that the synapsis and behaviour of *E.alaicus* (2n = 48) meiotic chromosomes were very similar to *E.tancrei* and *E.talpinus* ones (Kolomiets et al. 1991, 2010, Bakloushinskaya et al. 2012, Matveevsky et al. 2016, 2017). Isomorphic sex chromosomes exhibit a functional heteromorphism in the meiotic prophase I in all three species, that is a unique case for mammals.

Therefore, characteristic nucleotide substitutions in mitochondrial and nuclear genes, distinct Rbs variability and independent origin of typical for *E.alaicus* translocation Rb(2.11) support the species status of the Alay mole vole notwithstanding the closeness to *E.tancrei*.

The discovery of different heterozygous animals with 2n = 53 and two different Rb translocations raised the question of natural hybridization and mechanisms of genome stability. Animals that carried 1 Rb(2.11) with a high probability were hybrids of *E.alaicus*, 2n = 52 and *E.tancrei*, 2n = 54. For the second variant, 2n = 53 and 1 Rb(1.3), two scenarios are possible. The first is the existence of an unknown form (or species) with 2n = 52, 2 Rb(1.3), which hybridized with *E.tancrei*, 2n = 52, so hybrids of the first generation or backcrosses had 2n = 53, 1 Rb(1.3). Another possibility is that they were remote hybrids of *E.alaicus* with 2n = 50, 2 Rb(2.11), 2 Rb(1.3) (as animals from the Lake Chatyr-Kel’ vicinities, #4 or Naryn district, #6) and *E.tancrei*, 2n = 54. In that case, hybrids might have lost the Rb(2.11) in numerous generations under meiotic drive (de Villena and Sapienza 2001, Lindholm et al. 2016). Sociality described in mole voles (Smorkatcheva and Lukhtanov 2014, Smorkatcheva and Kuprina 2018) and underground lifestyle could accelerate the fixation of mutations in disjunct populations.

As we mentioned previously (Bogdanov et al. 2015), the differentiation of wide-ranging steppe species *E.talpinus* has occurred because of isolation due to geographic barriers, for example, large rivers such as the Volga River and the Irtysh River. *E.tancrei* and *E.alaicus* inhabit mountainous steppes and alpine meadows. Mountain ranges might be the most important geographic barriers for the spreading of mole voles because the animals do not inhabit mountains higher than 3500–4000 m above sea level. In the Tien Shan, the Pamir and the Pamir-Alay a distribution of mole voles should be sporadic because suitable habitats are mosaic. The complex orography of the regions may be a main source for geographical separation and ensuing fixation of the chromosomal forms (Bush et al. 1977). The situation is further complicated by the rapid change in the landscape due to neotectonic activity. The Alay Valley is an asymmetric intra-montane sedimentary basin with an average elevation of 2700 m, which formed in response to the convergence between India and Eurasia during the late Cenozoic (Coutand et al. 2002). The Pamir continues to move northward with a large fraction absorbed near the Alay Valley. The highest observed rate of the North-South convergence is between 10 and 15 mm/year as derived from Global Positioning System (GPS) measurements (Zubovich et al. 2016). The Pamir-Tien Shan region accommodates a high deformation over a short distance and is capable of producing magnitude 7 earthquakes in nearly decadal repeat times (Storchak et al. 2013). The last large seismic event was the 2008 magnitude 6.6 Nura earthquake with an epicenter just east of the Alay Valley (Sippl et al. 2014). Large earthquakes, which appeared to be in the Tien Shan and the Pamir, can trigger landslides (Havenith et al. 2003). Mudflows and landslides may quickly separate habitats of subterranean mole voles (Vorontsov and Lyapunova 1984). All three *E.alaicus* forms (2n = 52, 50 and 48) live in valleys, which are bordered by the mountain ranges. The evident pathways for mole voles spreading are the river banks in canyons crossing the ridges. Mole voles have a complex system of burrows, with at least three horizontal levels and numerous vertical connecting tunnels. But sometimes, most often at night, the animals run out onto the surface and move quickly over the ground. They probably can use human-made bridges, which are often destroyed by flows; new bridges may open a new route for mole voles. The suggestion was inspired when bursts of variations in chromosome numbers in mole voles from the opposite banks of the Vakhsh River were discovered at places close to bridges ([Bibr B45][Bibr B44]). In some cases, as when mole voles inhabit opposite banks of the Kyzyl-Suu River in a deep canyon (localities # 8, 9, Figs 1, 9, 10), we can only explain how animals cross a mountain river if we assume that they use human-made bridges.

**Figure 8.**
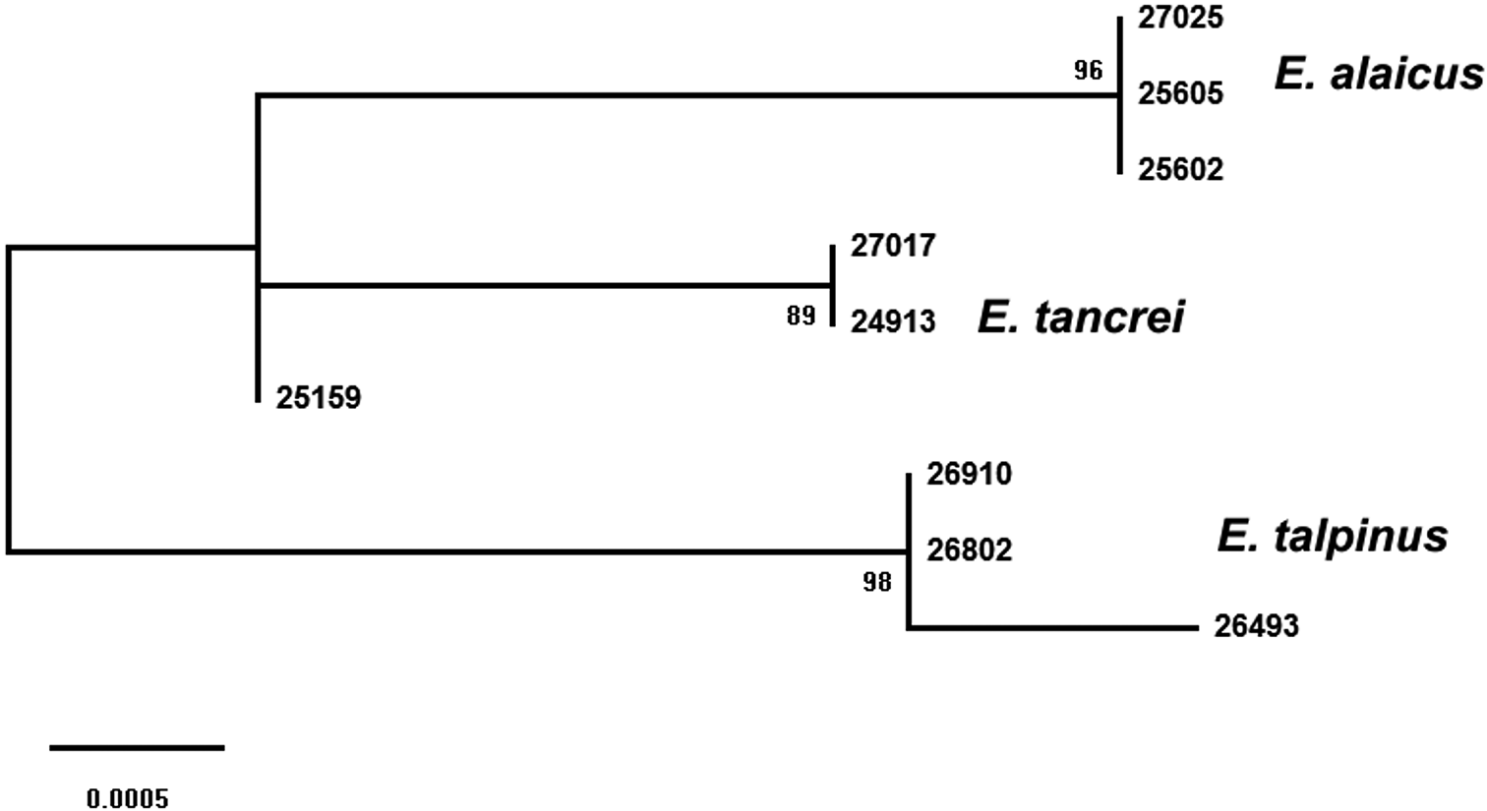
Molecular phylogenetic analysis of three *Ellobius* species based on variability of *XIST* and *Rspo1* genes fragments (1652 bp in total) and constructed by using the Maximum Likelihood method and the Jukes-Cantor model. Bootstrap support is listed for main branches. Only values over 70 percent are shown.

**Figure 9.**
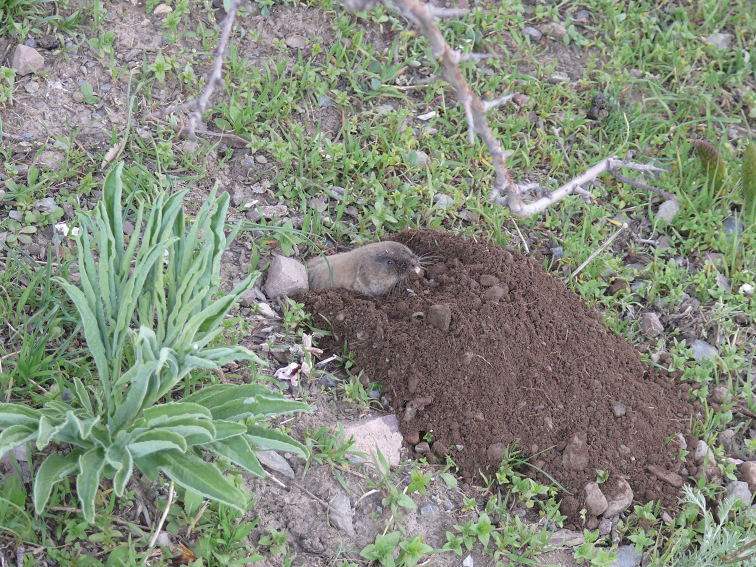
*Ellobiusalaicus*, locality #8. Photo by I. Bakloushinskaya.

Despite a complex relief of the region, the geographical barriers are not as strong as genomic ones. We revealed no signs of hybridization in neighbor populations of *E.alaicus* and *E.tancrei* yet, i.e. between *E.alaicus* (2n = 48, locality #8, Fig. 1) and *E.tancrei* (2n = 30, locality # 16, Fig. 1) or *E.alaicus* (2n = 48, locality #12, Fig. 1) and *E.tancrei* (2n = 54, locality # 13, Fig. 1). There are no geographical barriers preventing active contact between these populations in about ten or even few kilometers. In such cases, the assumption that genomic (chromosomal) reorganization in mammals is often rapid (Vorontsov, Lyapunova 1989, Bakloushinskaya 2016, Dobigny et al. 2017) seems plausible, if one considers that polymorphism for isolation traits segregates within populations with different genetic compositions and ecological settings. If we assume that loci, which may contribute to a reproductive barrier, are dispersed throughout the genome, and intragenomic interactions that arise from genetic pathways can maintain species-specific differences (Lindtke and Buerkle 2015, Payseur and Rieseberg 2016), we can consider speciation starting with chromosome changes as a reliable and fast way of speciation.

**Figure 10.**
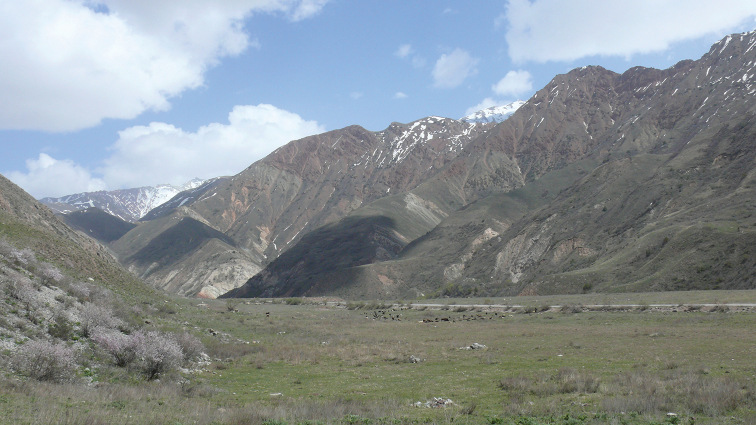
Habitat of *E.alaicus*, the Kyzyl-Suu River Valley, locality # 8. Photo by I. Bakloushinskaya.

## Conclusion

The study of *E.alaicus* demonstrates that the difficulty of species delimitation due to lack of morphological differences might be resolved by using chromosomal and molecular markers.

We assumed, that the independent emergence of Robertsonian translocation Rb(2.11) was crucial for the divergence of ancestors of *E.alaicus* and *E.tancrei*, which both developed specific karyotypic variability, more extensive in *E.tancrei* (2n = 54-30) but distinct due to non-homological (except Rb(2.11)) translocations in *E.alaicus* (2n = 52–48). Notwithstanding, the closeness of species, which was demonstrated here by studying mitochondrial DNA (*cytb*) and fragments of two nuclear genes, determines the possibility of sporadic hybridization at the zones of species contacts. Using different cytogenetic methods, G-banding and chromosome painting, along with by *cytb*, *XIST*, and *Rspo1* genes sequencing allowed us to expand the range of *E.alaicus* from the terra typica, the Alay Valley (South Kyrgyzstan) up to the Ferghana Ridge and the Naryn Basin, Tien Shan at the north-east and to the Pamir-Alay Mountains (Tajikistan) at the west.
